# How to Assess Diabetic Kidney Disease Progression? From Albuminuria to GFR

**DOI:** 10.3390/jcm10112505

**Published:** 2021-06-05

**Authors:** Clara García-Carro, Ander Vergara, Sheila Bermejo, María A. Azancot, Ana I. Sánchez-Fructuoso, M. Dolores Sánchez de la Nieta, Irene Agraz, María José Soler

**Affiliations:** 1 Nephrology Department, San Carlos Clinical University Hospital, 28040 Madrid, Spain; claragarciacarro@hotmail.com (C.G.-C.); sanchezfructuoso@gmail.com (A.I.S.-F.); sanchezdelanieta@senefro.org (M.D.S.d.l.N.); 2 Nephrology Research Group, Nephrology Department, Vall d’Hebrón Research Institute (VHIR), Vall d’Hebron Barcelona Hospital Campus, Vall d’Hebrón Hospital Universitari, 08035 Barcelona, Spain; avergara@vhebron.net (A.V.); sbermejo@vhebron.net (S.B.); maazancot@vhebron.net (M.A.A.)

**Keywords:** diabetic kidney disease, diabetes mellitus, chronic kidney disease

## Abstract

Diabetic kidney disease (DKD) is one of the most relevant complications of type 2 diabetes and dramatically increases the cardiovascular risk in these patients. Currently, DKD is severely infra-diagnosed, or its diagnosis is usually made at advanced stages of the disease. During the last decade, new drugs have demonstrated a beneficial effect in terms of cardiovascular and renal protection in type 2 diabetes, supporting the crucial role of an early DKD diagnosis to permit the use of new available therapeutic strategies. Moreover, cardiovascular and renal outcome trials, developed to study these new drugs, are based on diverse cardiovascular and renal simple and composite endpoints, which makes difficult their interpretation and the comparison between them. In this article, DKD diagnosis is reviewed, focusing on albuminuria and the recommendations for glomerular filtration rate measurement. Furthermore, cardiovascular and renal endpoints used in classical and recent cardiovascular outcome trials are assessed in a pragmatic way.

## 1. Introduction

In patients with type 2 diabetes, the prevalence of chronic kidney disease (CKD) is around 30–40%, mainly secondary to diabetic kidney disease (DKD) [1]. As for the general population, renal impairment increases the risk of cardiovascular (CV) disease for patients with type 2 diabetes [2]. This increased CV risk needs to be reduced in type 2 diabetic patients with CKD, because they have a baseline cardiovascular risk which is per se higher than that for the general population [3]. Even patients with stage 1 DKD have a cardiovascular risk comparable to that of patients with stages 2, 3, or 4 chronic kidney disease (CKD) caused by other diseases than diabetes [4]. For this reason, it is essential to slow down DKD progression to reduce mortality and morbidity in type 2 diabetes patients.

For decades, the only treatment that partially demonstrated to delay DKD progression has been renin–angiotensin system blockade. Nevertheless, in these last few years, new therapeutic options, such as sodium–glucose co-transporter 2 inhibitors (SGLT2i) and glucagon-like peptide-1 receptor agonists (GLP1a), have demonstrated a cardio-renal protective effect in type 2 diabetic patients [5,6,7]. The recommendation for these new drugs use has been implemented in several diabetes guidelines written by multidisciplinary teams composed of different specialist such as endocrinologists, nephrologists, internal medicine doctors, and cardiologists. These recommendations are based on cardiovascular outcome trials (CVOTs) that used different simple and composite endpoints, some cardiovascular and some other renal endpoints, difficult to interpret and to translate into the real-world clinical practice of general practitioners. In addition, the definition and diagnosis of DKD has not been clear for years, as well as the necessity of an annual renal evaluation of patients with diabetes. This could be the reason why patients are often referred to a nephrologist in advanced stages of DKD.

The aim of this article is to analyze available tools for DKD diagnosis, conduct a thorough review of the main cardiovascular and renal endpoints in CVOTs based on diabetic populations, and explore their utility in daily clinical practice. This review has mainly been based on a PubMed search and should be considered as narrative in nature.

## 2. General Considerations in Diabetic Kidney Disease

### 2.1. Diagnosis of DKD

The diagnosis of diabetic kidney disease (DKD) is based on clinical findings. It is defined by a decreased in glomerular filtration rate (GFR), the presence of albuminuria, or the existence of both dysfunctions in a patient with diabetes. A persistent reduction of estimated GFR below 60 mL/min/1.73 m^2^ and/or the existence of albuminuria (albumin-to/creatinine urine ratio ≥30 mg/g) in two measurements with at least a 3-month difference is sufficient to make a diagnosis of DKD in a patient with diabetes [8]. However, the term has a very low specificity, and a wide variety of histologic lesions, which are not always the consequence of diabetes, are usually included in this definition [9]. For instance, DKD could be misclassified in patients with obesity and hypertension, entities that commonly coexist. Therefore, the lack of precision of the term DKD raises another question: how could nephrologists be certain that diabetic lesions are the cause of CKD in their patients?

Kidney biopsy is the only way to confirm a diagnosis of kidney disease in a patient with diabetes and CKD, as well as the possibility of a non-diabetic renal disease diagnosis. This should always be done, especially when non-diabetic kidney disease (NDKD) is highly suspected. However, it is not always possible to perform a kidney biopsy in patients with diabetes, and thus, in these cases patients’ clinical history and clinical findings must be relied upon to guide the diagnosis of DKD. The sequence of events is a fundamental clue in this task (Table 1). The presence of CKD before the recognition of diabetes is normally associated with non-diabetic kidney disease (NDKD) [9]. The time since diabetes diagnosis is also relevant, especially in type 1 diabetic patients in whom it would be rare to identify albuminuria or diabetic kidney lesions with less than 5 years of disease duration [10,11,12]. On the contrary, in patients with diabetes and longer duration of the disease—known for more than 10 years—the most frequent lesions are diabetic nephropathy (DN) [13,14]. For both type 1 and type 2 DM patients, clinical findings such as the presence of progressive albuminuria before the decline of GFR or the diagnosis of diabetic retinopathy, also increase the probability of DN [15]. Conversely, the presence of hematuria has been related to NDKD [13,14].

Diabetic nephropathy, which is another essential term referring to diabetes complications, has been frequently used as a synonym of DKD [9,16]. Herein, the authors would like to point out that these concepts are not equivalent, and the use of the term DN should be restricted to patients with kidney biopsy-proven diabetic lesions. Therefore, DN is a more specific definition and confirms that diabetes is the actual cause of the pathological changes observed in the kidney. Diabetic kidney lesions create a pattern not usually seen in other renal diseases and sufficiently distinct to allow a diagnosis of DN [16]. The glomerulus is the most commonly affected structure in DN. Nevertheless, predominant tubulointerstitial damage with mild glomerular lesions is sometimes seen in diabetic patients and is related to the renal prognosis. In type 2 diabetes, where pathological changes result from multiple comorbidities, lesions are more heterogeneous, and tubulointerstitial damage may be predominant over glomerular injury [16]. In 2010, Tervaert et al. published a consensus classification of DN to establish the severity of these pathological changes [17]. Subsequently, a kidney biopsy, in addition to increasing diagnostic accuracy, provides information about the severity and reversibility of kidney lesions. Interestingly, the presence of renal impairment in diabetic patients, related to demonstrated ND or clinical suspected DKD, increases their cardiovascular risk. For this reason, new strategies to promote the early diagnosis and treatment of renal injury in patients with diabetes are urgent in nephrology clinical research nowadays.

### 2.2. Progression of DKD

**Figure 1:**
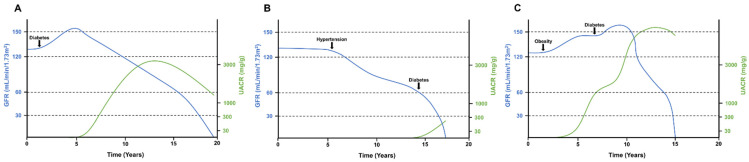
Glomerular filtration rate and albuminuria over time in three different hypothetical situations of DKD. GFR decline and albuminuria can vary in patients with DKD depending on previous comorbidities and the timing of harmful events. (**A**) Classical phenotype of DKD with an initial hyperfiltration phase and later development of progressive albuminuria. As the disease advanced to overt nephropathy, GFR decline was observed. (**B**) Non-proteinuric DKD in a patient that had hypertension before a diabetes diagnosis. Note that mild albuminuria appeared only when the patient had advanced CKD. (**C**) Glomerular hyperfiltration in an obese patient that later developed diabetes. As hyperfiltration progressed, the patient developed massive albuminuria followed by a rapid decline in GFR. UACR: urinary albumin-to-creatinine ratio. GFR: glomerular filtration rate.

In type 1 diabetic patients, it is possible to establish a precise date for the onset of insulin dependence, as well as for the diagnosis of the disease. Therefore, the clinical course of DKD was traditionally described in type 1 diabetic patients [18,19]. In these patients, a first silent phase of glomerular hyperfiltration is followed by mild albuminuria (urinary albumin of 30 to 300 mg/day). After an average of 10–15 years from diagnosis, albuminuria progresses to overt proteinuria, and reduction of GFR begins (Figure 1) [19,20]. However, as the knowledge of DKD improved, it has been observed that not all diabetic patients present the classic phenotype. Many patients with diabetes, especially the heterogeneous group of type 2 diabetic patients, have a decline in GFR without albuminuria [15].

Different factors may influence the speed at which GFR declines in DKD. Other comorbidities that develop both before and after diabetes onset, such as obesity, hypertension, or dyslipidemia, could contribute to an accelerated reduction of GFR [15]. Furthermore, acute kidney injury episodes or the development of DKD over a previously known CKD accelerate the evolution to end-stage kidney disease (ESKD) [9]. The risk factors associated with GFR decline in DKD are hypertension, obesity, and dyslipidemia [15,21]. Besides, it is worthy of mention that a GFR decline in diabetes can even occur in the absence of albuminuria [15]. As previously mentioned, a considerable number of patients with diabetes have predominant interstitial lesions with little or no glomerular damage. Thus, the renal function could be impaired without glomerular injury and the consequent development of albuminuria [10].

### 2.3. Albuminuria in DKD

Albuminuria has been classically considered the first DKD clinical indicator, a biomarker for DKD progression, and a cause of impairment of GFR [22] (Table 2). As stated above, the presence of albuminuria A2 in a diabetic patient confirmed by two measurements is enough for a DKD diagnosis; however, its presence is not only a static clinical marker. Untreated albuminuria will gradually worsen, turning into clinical severe albuminuria grade A3 (albumin-to-creatinine urine ratio >300 mg/g) at 10–15 years after diabetes diagnosis. The prevalence of albuminuria grade A3 in type 2 diabetes ranges from 5% to 48% depending on the studies, and in type 1 diabetes, from 8% to 22%. The albuminuria grade A2 presence in diabetic type 1 and type 2 patients is 13% and 20%, respectively [23]. In some cases, albuminuria may regress, either spontaneously or in relation to treatment, resulting in a lower renal risk in these patients when compared with patients who present progression of albuminuria. On the other hand, the presence of impaired GFR in the absence of albuminuria in diabetic patients, mainly in elderly populations, confers a lower risk of progression to ESKD [24].

**Figure 2:**
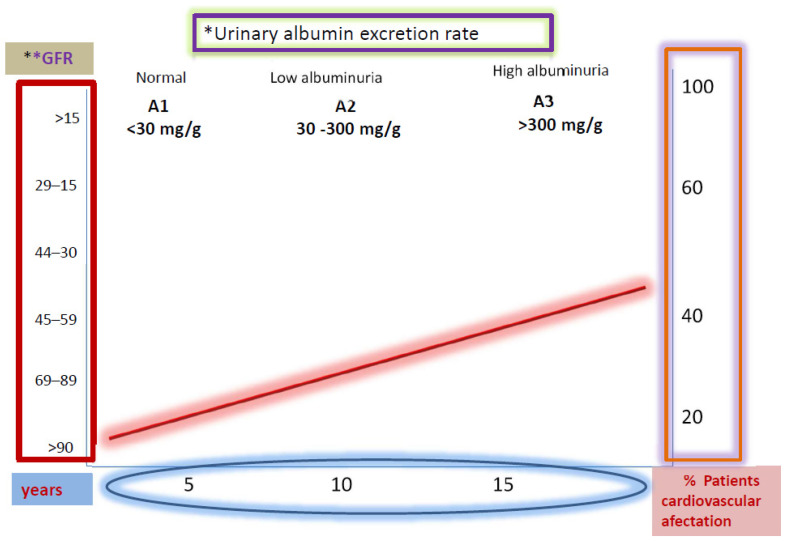
Correlation between renal function deterioration and degree of diabetic nephropathy. UAER: * Urinary albumin excretion rate: mg/gr creatinine; ** GFR mL/min/1.73 m^2^.

Albuminuria is considered an independent risk factor for cardiovascular disease, and a higher rate of urinary albumin excretion is associated with an increased incidence of cardiovascular morbidity and mortality, as shown in Figure 2 [26,27]. Currently, an annual screening to detect abnormal levels of albuminuria and renal function measurement has been recommended by the National Kidney Foundation Kidney Disease (KDOQI) working group practice guideline in patients with diabetes.

It is also recommended to initiate a renoprotective treatment in the early stages of DKD. Moreover, for the evaluation of GFR in diabetic patients, the recommendation is to use a creatinine-based formula such as the Chronic Kidney Disease Epidemiology Collaboration (CKD-EPI) equation [28]. The classification of DKD based on albuminuria and GFR give us prognostic information and helps us to make adequate therapeutic decisions. In clinical practice, the adherence to guidelines regarding albuminuria screening and treatment recommendations is not very high, as demonstrated by the GIANTT trial [29]. One of the reasons for this lack of adherence to screening could be that methods for albuminuria assessment are not standardized. The chosen measurement method for every single patient should be the one repeated over time to detect as early as possible the progression of DKD [30].

The presence of albuminuria, however, is a quite late indicator of DKD. As soon as albuminuria is detected, kidney injury is already established. In the nearly future, new circulating and urinary biomarkers, identified by genomics, transcriptomics, metabolomics, and proteomics, are needed to perform an earlier diagnosis of renal risk in diabetes and to improve the renal and global prognoses in these patients.

## 3. Cardiovascular Endpoints in Diabetic Kidney Disease

As mentioned above, patients with type 2 diabetes have a higher prevalence of cardiovascular morbidity and mortality as compared to the general population. The presence of kidney involvement in patients with cardiovascular disease, especially in patients with diabetes, confers an unfavorable prognostic and an increased cardiovascular risk. Kidney dysfunction in patients with diabetes is a marker of vascular lesions, and their detection allows the early identification of individuals at high risk for cardiovascular events. This early detection is necessary to improve their prognosis [25].

During the early stages of diabetes, there is an increase in plasma renin activity that plays a major role in the development of cardiovascular disease. Classically, angiotensin-converting enzyme inhibitors (ACEi) and angiotensin II receptor blockers (ARB) have demonstrated effectiveness in reducing renal progression and mortality in patients with diabetes and renal disease [31]. For decades, metformin and sulfonylureas have been the first-line drugs for managing type 2 diabetes, and their use in patients with eGFR between 45–60 mL/min has shown a reduction in mortality from all causes [32]. In a large cohort study (*n* = 124,720), Christiansen et al. [33] demonstrated that patients who started a treatment with metformin had a lower risk of a severe decrease in GFR. However, the use of metformin has been associated with lactic acidosis in the context of acute kidney injury [34], although a good renal prognosis has also been shown [35]. Furthermore, in patients under treatment with sulfonylureas or metformin, the addition of pioglitazone or acarbose was not able to demonstrate changes in the evolution of renal function or ACR [36]. It is of note that the use of sulfonylureas in patients with reduced GFR increases the risk of hypoglycemia [37].

During the last years, several studies have placed new antidiabetic drug families, such as sodium-glucose cotransporter-2 inhibitors (SGLT2i) and glucagon-like peptide 1 agonists (GLP1a), on top of classical treatments, making them the new first-line therapies in the prevention of cardiovascular events in this population [38]. These new classes of drugs have been tested in multiple CVOTs that have shown positive results in terms of cardiovascular risk. However, there is wide variability in the specific cardiovascular outcomes assessed in every trial. This disparity in the endpoints makes the comparison between them difficult. The scientific community has the necessity to establish relevant cardiovascular variables in the follow-up of patients with type 2 diabetes in CVOTs and to redefine which variables have repercussions on a real-world scenario [38,39].

Classical studies for ACEi and ARB analyzed primary cardiovascular outcomes such as mortality and hospitalization for congestive heart failure or a combined outcome of both as in the SOLV trial [40]. A few years later, in the HOPE trial [31], the primary outcome was already identified as a composite cardiovascular one with myocardial infarction, stroke, or death from cardiovascular causes, and each of these outcomes was also analyzed separately.

MACE (Major Adverse Cardiovascular Event) has been described and identified as the primary outcome in the vast majority of CVOTs involving patients with diabetes in this last decade [41]. It is a combined clinical endpoint used for cardiovascular outcome evaluations in CVOTs and it is comparable to the composite endpoint of all-cause mortality. The so-called classical 3-point MACE (3pMACE) is defined as a composite of death from a cardiovascular cause, nonfatal myocardial infarction, and nonfatal stroke. More recent studies have assessed a 4-point MACE (4pMACE) that includes hospitalization for unstable angina and/or a 5-point MACE that adds hospitalization for heart failure.

CVOTs of new antidiabetic agents have been taking the MACE term into account (Table 3). Most dipeptidyl-peptidase 4 inhibitors (iDPP-4) clinical trials used a 3pMACE, except for the TECOS [42] trial that used a 4pMACE. In this group of CVOTs, only the CARMELINA [43] trial analyzed a secondary renal outcome. On its behalf, the GLP1a clinical trials (LEADER [6], SUSTAIN-6 [44], HARMONY [45], REWIND [46], EXSCEL [47] and PIONEER [48]) used a 3pMACE, while more recent trials such as ELIXA [49] and FREEDOM-CVO [50] used a 4pMACE 4. Interestingly, five out of eight GLP1a studies analyzed kidney variables as outcomes. The SGLT2i trials (EMPA-REG [5], CANVAS [51], DECLARE-TIMI 53 [52], VERTIS CV [53], and CREDENCE [54]) used a 3pMACE, but all of them evaluated a renal endpoint, being in the CREDENCE study a primary renal endpoint.

As MACE and its variations are a good strategy for a clinical comparison of CVOTs, so the inclusion of renal outcomes in CVOTs with antidiabetic drugs is fundamental for the rational evaluation of patients with type 2 diabetes.

## 4. Composite Renal Outcomes

In most clinical trials evaluating complications of type 2 diabetes, composite endpoints have been used, as previously mentioned. Decreases in sample size requirements and the ability to assess the net effect of an intervention and to avoid bias in the presence of competing risks are the most cited advantages for their use. The vast majority of clinical trials in type 2 diabetes used cardiovascular criteria as primary composite outcome and renal endpoints as secondary pre-specified objectives [5,58]. Secondary endpoints are additional endpoints, for which the trial may not be powered. The US Food and Drug Administration (FDA) indicated that secondary endpoint measures, by themselves, are not sufficient to fully characterize a treatment benefit. However, these measures may provide additional characterization of the treatment effect. Moreover, in several studies, the renal effects were evaluated in post-hoc analysis and not predefined in the protocol [56,59].

Recently, three seminal studies, CREDENCE [54],DAPA-CKD [60], and FIDELIO-DKD [57], have been published in the field of DKD, with renal outcomes as primary studies endpoints. These trials are different, but all analyzed the onset, the worsening of nephropathy, and patient’s death due to renal causes. Heterogenicity in the endpoints has become a big problem to solve when comparing trials. For example, to evaluate the impairment of renal function, different parameters have been proposed, including a decrease in GFR greater than 30, 40, or 50% or the doubling serum creatinine [54,58,59,60]. The same problem had arisen with other essential variables such as albuminuria and the definitions of ESKD and death from renal causes. Diverse effects, such as hemodynamics, can temporarily alter the creatinine and albuminuria values, so it remains mandatory to repeat and verify these parameters in the clinical evaluation of a diabetic patient.

For all the above-mentioned reasons, it is mandatory to define uniform criteria applicable to the design of clinical trials to be conducted in the next future. In this sense, a unifying definition of renal outcomes has been proposed combining three to five major adverse renal events (MARE). Recently, it has been used in clinical studies in patients with diabetes. MARE has been defined as: 1) incident kidney disease determined as new onset of kidney injury measurable by sustained eGFR < 60 mL/min/1.73 m^2^ (on three consecutive visits) and/or new onset of albuminuria (UACR > 30 mg/g on at least two of three measurements on three consecutive days); 2) worsening of kidney disease determined as a sustained > 40% reduction in GFR or slope of GFR based on at least seven creatinine measurements and resulting in a significant GFR decline over a time period to be defined (most likely two years) and/or a slope (significant increase) in UACR compared with baseline measured in at least two of three urine samples on three consecutive days, confirmed by a second three-day urine measurement at least one month from the first result; 3) ESKD determined by initiation of renal replacement therapy (RRT) and continued for at least three months (or refusal of the patient or inability to start RRT for other reasons); 4) death due to renal causes determined as the death directly attributable to kidney disease (hyperkalemia and death from arrhythmia, calciphylaxis aggravating CV disease and subsequent death, or decompensated heart failure not explained by acute myocardial ischemia and death from uremia); 5) death of non-renal cause defined as death of any origin excluding kidney disease. Additionally, patient reported outcomes should be reported in parallel to MARE as a standard set of endpoints in studies on kidney disease in patients with diabetes.

## 5. GFR Decline in DKD: Ways of Measurement and Threshold

The development of CKD in diabetic patients is one of the most important complications in this population. Furthermore, DKD is the first known cause of CKD in developed countries [61]. Renal damage in diabetics is not only in the glomerular compartment but also in the tubulo-interstitial and vascular compartments. In clinical practice, a decrease in renal function and the presence of albuminuria have been considered as markers of renal damage [8,62]. These variables are used in daily clinical practice for screening. The classic method of measuring kidney function is the determination of the plasma creatinine level. However, it is not the best method, since it depends on multiple variables, such as muscle mass, age, sex, race, etc. For this reason, even in the presence of a normal creatinine level, it is possible that a reduction in nephrons function already exists, indicated by a decrease in GFR [39]. There are two ways to obtain the GFR: a direct measurement and an indirect calculation. Regarding the direct measurement of GFR, it is possible to use a technique based on radioisotopes or radiopharmaceuticals, not useful in routine clinical practice. Another method for the direct determination of GFR requires calculating creatinine clearance in 24 h urine. However, since creatinine excretion at the urinary level can be altered by many factors, an overestimation of the GFR is possible, and this method does not offer many advantages compared to the indirect calculation. Therefore, the indirect calculation has been widely recommended as a routine screening method [28]. Regarding the indirect calculation of GFR, different formulas derived from serum creatinine levels have been applied, such as the Cockcroft–Gault, MDRD-4, and CKD-EPI ones. Currently, CKD-EPI is the method widely recommended in clinical practice guidelines [25,63]. As it is known, CKD in diabetic patients corresponds to a decrease in GFR < 60 mL/min and / or the presence of microalbuminuria for more than three months [25]. However, in the evolution of DKD, there exists a first phase of hyperfiltration that is hard to diagnose, where there is an increase of GFR without albuminuria secondary to glomerular hyperfiltration [64]. Without treatment, the natural history of DKD leads to the loss of renal function with a decrease of between 2 and 20 mL/min of eGFR per year. However, with adequate glycemic control, blood pressure treatment, reduction of cholesterol levels, hygienic–dietary measures, and lifestyle changes, the loss of renal function may be substantially delayed, with a decrease of GFR between 2 to 5 mL/min per year [30]. In the evaluation of GFR as a renal endpoint in clinical trials, the doubling of the serum creatinine level has been classically used. This decrease corresponds to a reduction in GFR by 57% [65], indicating that this is a late marker, and although it is strongly related to CKD progression, large cohorts and long follow-up periods are needed to obtain this endpoint. For this reason, lower percentages of reduction of the GFR have been recently used in clinical trials analyzing renal outcomes, as previously described, such as GFR reduction of 40 or 30% [65]. An interesting paper by Perkovic et al. was designed to assess the consistency of the effects of empagliflozin versus placebo on an alternative composite kidney endpoint, consisting of different thresholds of decline in eGFR, initiation of renal replacement therapy (RRT), or renal death in the EMPA-REG OUTCOME trial, to assist in the design of future kidney trials. This study demonstrated that empagliflozin consistently reduced the risk of a broad range of kidney composite outcomes using different eGFR thresholds (≥30%, ≥40%, ≥50%, and ≥67%) to define a significant loss of kidney function. In addition, this study suggests that the use of a composite endpoint consisting of a 40% decline in eGFR, ESKD, and renal death may be the most reliable and efficient choice to demonstrate clear kidney benefits with the smallest required sample size and the greatest study power, when sustained outcomes are used [66].

## 6. Pros and Cons of Renal Endpoints Standardization

DKD is closely associated with a significant increase in CV risk. Early detection of kidney disease is of vital importance to stratify patients at risk for CV morbidity and mortality and to improve their prognosis by initiation of several treatments reflected in the current clinical practice guidelines [25]. Despite the fact that DKD is frequent and associated with an increase of mortality and patient burden cost, in different studies, the definition of renal outcomes is variable and heterogeneous, as previously mentioned in this review. Nephrology-oriented research is insufficient to answer the large amount of paramount clinical questions [67]. Nephrology is a specialty in which few randomized clinical trials have been carried out [68,69], and this is particularly true for studies evaluating glucose-lowering drugs and the risk of DKD progression.

In 2008, the FDA published its guidelines to support the pharmaceutical industry in CVOTs for the development of new type 2 antidiabetic drugs, albeit renal outcomes were often evaluated as secondary endpoints in this CVOTs. Several studies focused on the renal effects of specific antidiabetics drugs, for example, on GFR or albuminuria trends, using heterogeneous defined endpoints, which makes difficult the interpretation of the results and their applicability in clinical practice [70].

During the last 15 years, several classes of antidiabetics drugs have been introduced for the treatment of patients with diabetes, including SGLT-2i, DPP-4i, and GLP-1a, and interestingly, secondary outcomes reported by CVOTs have indicated that these drugs may directly improve the renal function beyond changes in glycemic control. Recently, the SGLT2i family has emerged as a major advance for the treatment of DKD. The results of the CREDENCE trial have undoubtedly demonstrated that canagliflozin prevents kidney failure and cardiovascular events [71]. In addition to these three groups of drugs previously mentioned, the FIDELIO-DKD study showed that the use of finerenone (nonsteroidal, selective mineralocorticoid receptor antagonist) resulted in a lower risk of CKD progression and cardiovascular events compared to the use of placebo. In this case, the composite primary renal endpoint was kidney failure, sustained ≥ 40% decrease in GFR from baseline, or death from renal causes. Finererone was superior to placebo in these composite primary renal outcomes and also in the secondary composed outcome of reduced ACR [57]. These are the most important studies that assessed a composite renal endpoint as its primary endpoint and showed positive effects on these hard renal outcomes. In this regard, MARE, comparable to the term MACE, has been described to evaluate in a homogeneous way several events related to the development of new-onset DKD, ESKD, mortality, and quality of life [70]. With this new proposal, the homogenization of renal primary outcomes probably will help in the next future to respond to several questions on the management of DKD and the risk of progression. However, this approach may generate some doubts in CVOTs with composite endpoints. Regardless of this, a uniformly agreed definition for MARE would make meta-analyses easier and would facilitate the comparison of different studies, allowing tailored treatments for patients with diabetes at risk for ESKD.

## 7. Conclusions

For physicians involved in the management of patients with diabetes, it is crucial to understand the importance of the diagnosis of DKD. This diagnosis is easy and cheap to achieve by measuring GFR and UACR and is the first step to prevent DKD progression. Albuminuria is a good biomarker but reflects the presence of established kidney damage. Thus, hyperfiltration before albuminuria appearance must warn the clinician to start therapeutic adjustments for renal and cardiovascular protection. This new therapeutic approach for type 2 diabetes is based on CVOTs in diabetic populations with several cardiovascular and renal endpoints. The differences between endpoints in CVOTs make difficult the comparison of outcomes. However, the establishment of MACE is a first step towards a clarification; similarly, MARE describes for first-time the renal endpoints and allows the scientific community to design new clinical trials focused on renal involvement in type 2 diabetes.

## Figures and Tables

**Table 1:** Predictive risk factors for diabetic and non-diabetic kidney disease. Clinical findings that may help to identify diabetic and non-diabetic kidney disease in patients with diabetes and CKD.

Predictive Risk Factors for DKD	Predictive Risk Factors for NDKD
-Diabetic retinopathy	-Microhaematuria
-Longer duration of DM (≥10 years)	-Shorter duration of DM (<5 years)
-Chronic lower limb ischemia	-Overweight grade II (BMI: 35–39.9 kg/m^2^).
-Nephrotic range proteinuria	-Older age
-Insulin treatment	-History of CKD prior to diabetes development

DKD: diabetic kidney disease. NDKD: non-diabetic kidney disease. DM: diabetes mellitus. CKD: chronic kidney disease, BMI: Body Mass Index.

**Table 2:** Classification of persistent albuminuria. Adapted from KDIGO Guidelines [25] ACR: Albumin/creatinine ratio. AER: Albumin excretion rate.

Category	AER	ACR	ACR	Renal Composite Outcome
	(mg/24 h)	(mg/mmol)	(mg/g)	Terms
A1	<30	<3	<30	Normal to mildly increased
A2	30–300	3–30	30–300	Moderately increased
A3	>300	>30	>300	Severely increased

**Table 3:** Principal baseline characteristics and major renal outcomes of interest in the recently published type 2 diabetes outcome trials.

Trial	Drug	Population	Baseline Renal Characteristics	Renal Composite Outcome	Albuminuria	GFR/Creatinine	ESRD
EMPA-REG OUTCOME [5]	Empagliflozin	7020 patients with T2D with GFR > 30 mL/min	25.9% of patients had GFR <60 mL/min	Progression to macroalbuminuria, doubling of serum creatinine with GFR ≤ 45 mL/min, ESRD, renal death	Incident microalbuminuria (UACR 30–300 mg/g) Incident macroalbuminuria (UACR > 300 mg/gr)	Doubling of serum creatinine and GFR < 45 mL/min	Need of RRT
CANVAS program [51]	Canagliflozin	10142 patients with T2D and cardiovascular disease.	20.1% of patients had GFR < 60 mL/min	Sustained ≥ 40% decrease in GFR, ESRD or renal death	New microalbuminuria or new macroalbuminuria with ≥ 30% increased UACR	Sustained 40% reduction in GFR for ≥ 30 days	Sustained GFR < 15 mL/min for > 30 days, dyalisis ≥ 30 days or renal transplant.
CREDENCE trial [54]	Canagliflozin	4401 patients with T2D, GFR 30–89 mL/min and UACR 300–5000 mg/gr	All patients had GFR of 30–89 mL/min and UACR 300–5000 mg/g	Doubling of serum creatinine, ESRD, or death from renal or cardiovascular disease	Comparison of UACR versus placebo	Sustained doubling of serum creatinine	Sutained GFR < 15 mL/min for > 30 days or need for dialysis or renal transplant
DECLARE-TIMI 58 [52]	Dapagliflozin	17160 patients with T2D and cardiovascular disease	7.4% of patients had GFR ≤ 60 mL/min	Sustained ≥ 40% decrease in GFR to ≤ 60 mL/min, ESRD, renal or cardiovascular death	Comparison of UACR versus placebo	Sustained ≥ 40% decrease in FGR to ≤ 60 mL/min	Sustained GFR < 15 mL/min, or dialysis for ≥ 90 days or renal transplant
DAPA-HF [55]	Dapagliflozin	4744 patients with T2D and non-T2D, HF with EF < 40%	All patients had GFR > 30 mL/min	Sustained ≥ 50% decrease in GFR, ESRD, renal death	Not reported	Sustained ≥ 50% decrease in GFR	Sustained GFR < 15 mL/min ≥ 28 days, or need for continuous RRT
SAVOR-TIMI [56]	Saxagliptin	16492 patients with T2D and cardiovascular disease	15.6% of patients had GFR < 50 mL/min	Doubling serum creatinine or ESRD	Categorical change in UACR from baseline	Doubling of serum creatinine	Need for renal dialysis, transplant or serum creatinine > 530 µmol/L
CARMELINA trial [43]	Linagliptin	6979 patients with T2D and high cardiovascular and renal risk	74% of patients had GFR of 30–59 mL/min and 15.2% had GFR < 30 mL/min	Sustained ≥ 40% decrease in GFR and GFR ≤ 60 mL/min, ESRD, renal death	Microalbuminuria (ACR 30–300 mg/gr) or macroalbuminuria (UACR ≥ 300 mg/g)	Sustained ≥ 40% decrease in GFR and GFR ≤ 60 mL/min	Need for renal dialysis ≥ 30 days or renal transplant
LEADER trial [6]	Liraglutide	9340 patients with T2D	23.1% of patients had GFR < 60 mL/min	New macroalbuminuria, doubling of serum creatinine with GFR ≤ 45 mL/min, need for continuous RRT or renal death	New macroalbuminuria (UACR > 300 mg/gr or urinary albumin > 300 mg/24 h)	Doubling of the serum creatinine with GFR ≤ 45 mL/min	Need for continuous RRT
SUSTAIN-6 trial [44]	Semaglutide	3297 patients with T2D and Cardiovascular disease	28.5% of patients had FGR < 60 mL/min	New macroalbuminuria, doubling of serum creatinine with GFR ≤ 45 mL/min, need for continuous RRT or renal death	New macroalbuminuria (UACR > 300 mg/gr or urinary albumin > 300 mg/24 h)	Doubling of the serum creatinine with GFR ≤ 45 mL/min	Need for continuous RRT
EXSCEL trial [47]	Exenatide	14752 patients with T2D	21.6% of patients had GFR < 60 mL/min	New macroalbuminuria sustained ≥ 40% decrease in GFR or RRT or renal death	New macroalbuminuria	Sustained ≥ 40% decrease in GFR	Need for continuous RRT
REWIND study [46]	Dulaglutide	9901 patients with T2D	22.2% of patients had GFR < 60 mL/min	New macroalbuminuria, sustained ≥ 30% decrease in GFR or chronic RRT	New macroalbuminuria (UACR > 33.9 mg/mmol)	Sustained ≥ 30% decrease in GFR	Need for continuous RRT
FIDELIO-DKD [57]	Finerenone	5734 patients with CKD and T2D	All patients had GFR of 25–60 mL/min and UACR of 300–5000 mg/g	Kidney failure, sustained ≥ 40% decrease in GFR or death from renal causes	Change in UACR from baseline to month 4	Sustained ≥ 40% decrease in GFR	GFR < 15 mL/min or initiation of RRT (≥ 90 days) or kidney transplantation

GFR: glomerular filtration rate; ESRD: end-stage renal disease; UACR: urinary albumin/creatinine ratio; T2D: type 2 diabetes; RRT: renal replacement therapy.
